# Supplementary material for: Dancing the Nanopore limbo – Nanopore metagenomics from small DNA quantities for bacterial genome reconstruction
Source: BMC Genomics. 2023 Dec 1;24:727. doi: 10.1186/s12864-023-09853-w (PMC10693096; doi:10.1186/s12864-023-09853-w)
Supplement: Supplementary file 2 — Supplementary Material 2 [file 12864_2023_9853_MOESM2_ESM.docx]

*Supplements for*

**Dancing the Nanopore limbo – Nanopore metagenomics from small DNA quantities for bacterial genome reconstruction**

Sophie A. Simon^1^, Katharina Schmidt^1^, Lea Griesdorn^1^, André R. Soares^1^,

Till L. V. Bornemann^1^ and Alexander J. Probst^1,2^

1: Environmental Metagenomics, Research Center One Health Ruhr of the University Alliance Ruhr, Faculty of Chemistry, University of Duisburg-Essen

2: Centre of Water and Environmental Research (ZWU), University of Duisburg-Essen, Essen, Germany

To whom the correspondence should be addressed: sophie.simon@uni-due.de and alexander.probst@uni-due.de

List of content:

1. Supplementary Tables
2. Supplementary Figure
3. **Supplementary Tables**

All tables (Table S1-S5) are provided as a separate file <supplement_tables.xlsx>

1. **Supplementary Figures**

**
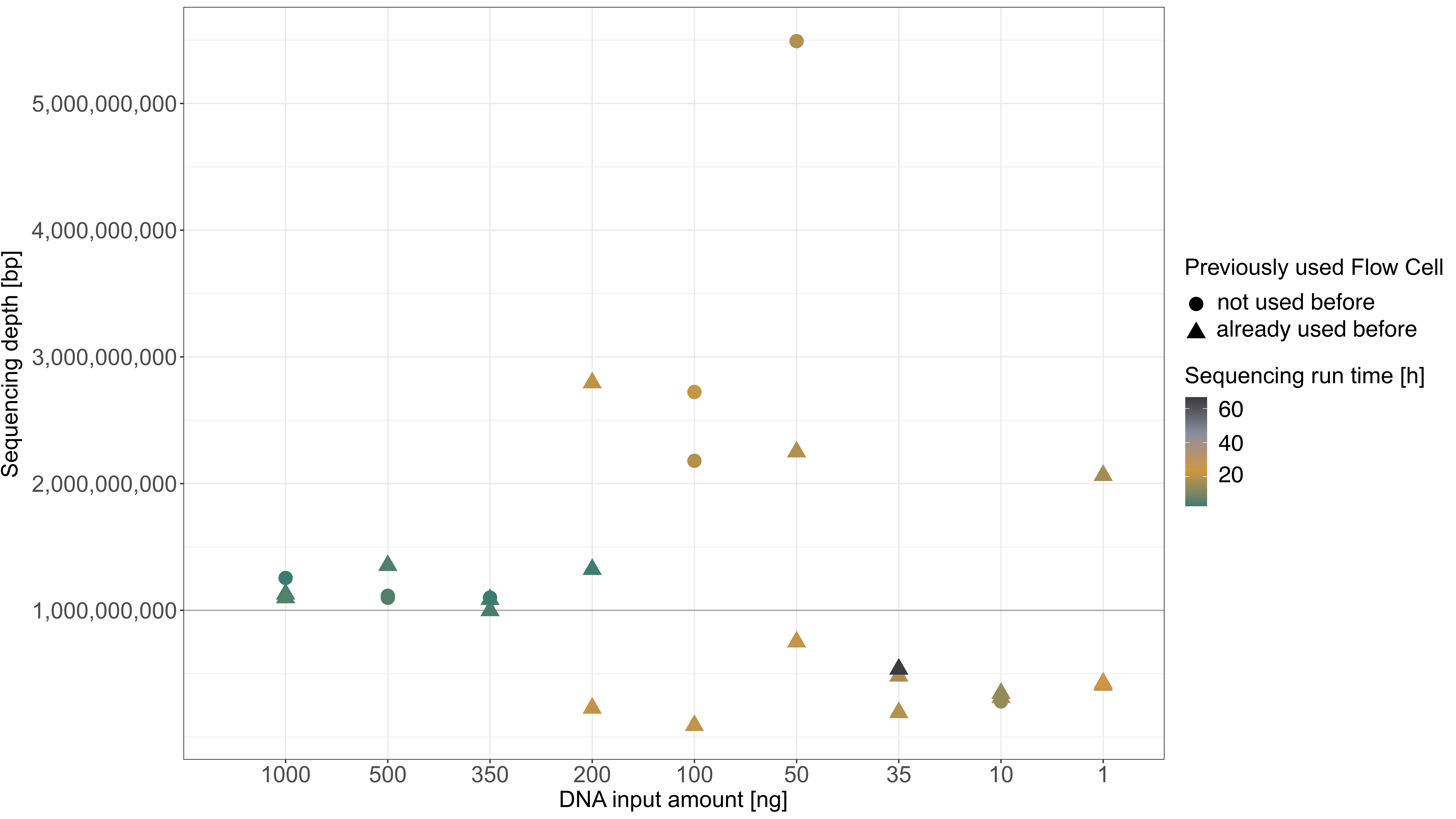
**

**Figure S1 |** Sequencing depth in terms of Flow Cell usage and sequencing run time. Aiming for 1 Gb in total we manually stopped sequencing runs as soon as 1 Gb was achieved. Outliers towards a higher sequencing depth were sequenced overnight and thus stopped later as necessary. Outlier towards a lower sequencing depth were stopped after the sequencing output plateaued. The shape indicates if a Flow Cell has been used and washed before the respective sequencing run.


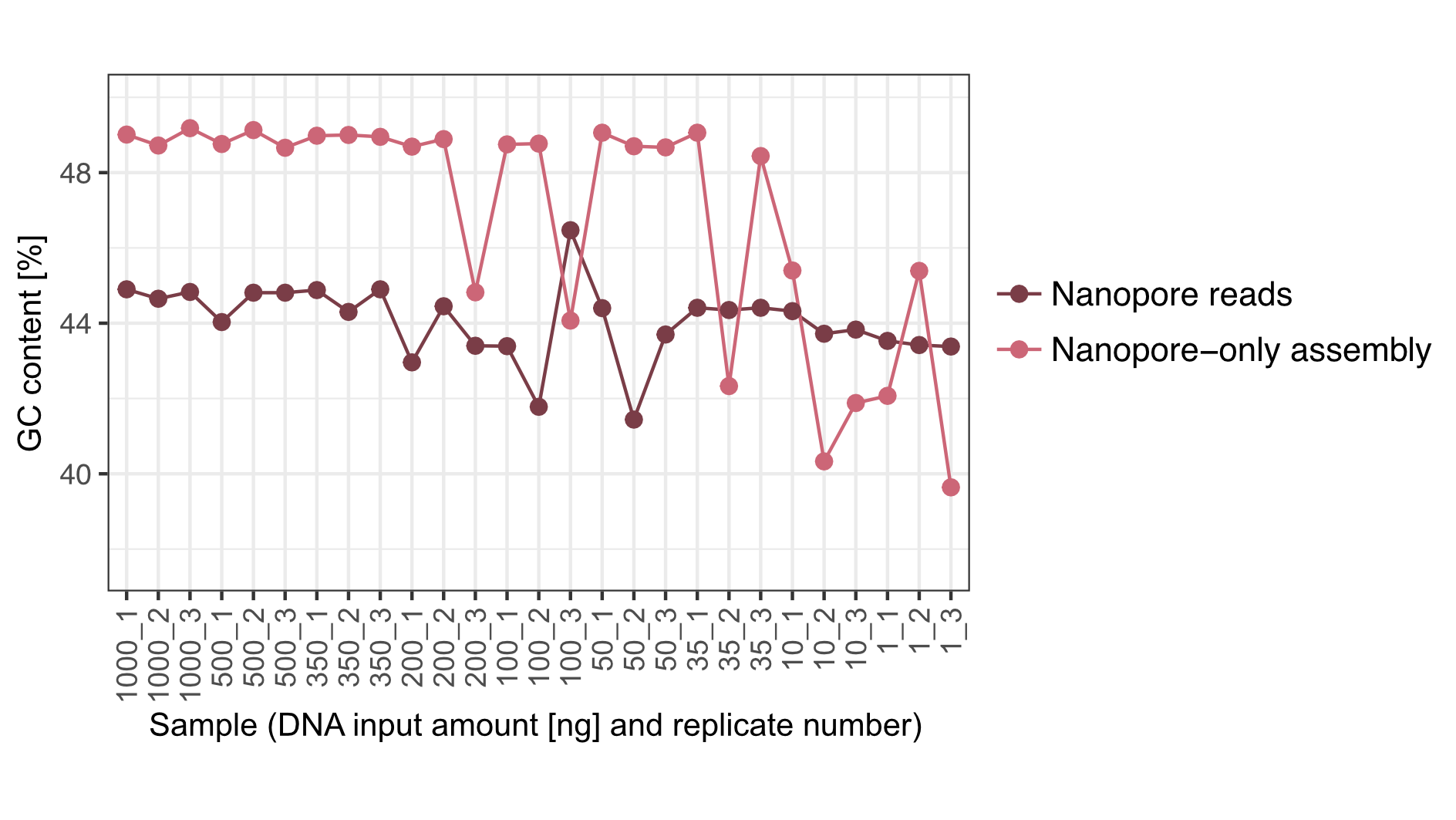


Figure S2 | GC-content [%] assessed for Nanopore reads and Nanopore-only assemblies per sample. The GC-content varies distinctly for assemblies of less than 50 ng DNA input, while the GC content of the reads itself remains fairly stable. The GC content of the yeast *Saccharomyces cerevisiae* is given as 38.3% (https://files.zymoresearch.com/protocols/_d6322_zymobiomics_hmw_dna_standard.pdf, [26.01.23]), since it is not assembled by metaFlye, it is expected that GC content of the reads lower than of the assemblies.
